# The fibrotic microenvironment as a heterogeneity facet of hepatocellular carcinoma

**DOI:** 10.1186/1755-1536-6-17

**Published:** 2013-09-16

**Authors:** Krista Rombouts, Vinicio Carloni

**Affiliations:** 1 Institute for Liver and Digestive Health, Royal Free Hospital, University College London UCL, London, UK; 2 Department of Experimental and Clinical Medicine, Center for Research, Transfer and High Education, DENOthe, University of Florence, Largo Brambilla 3, 50134, Florence, Italy

**Keywords:** Liver cancer, Activated hepatic stellate cells (HSC), Intra-tumor heterogeneity, Tetraspanin-enriched microdomains, Integrins

## Abstract

It has long been recognized that hepatocellular carcinoma heterogeneity arises from variation in the microenvironment or from genomic alteration. Only recently it has become clear that non-genetic alterations, such as cytoskeletal rearrangement, protein localization and formation of protein complexes, are also involved in generating phenotype variability. These proteome fluctuations cause genetically identical cells to vary significantly in their responsiveness to microenvironment stimuli. In the cirrhotic liver pre-malignant hepatocytes are continuously exposed to abnormal microenvironments, such as direct contact with activated hepatic stellate cells (HSCs) and extracellular matrix components. These abnormal environments can have pronounced influences on the epigenetic aspects of cells, translating into abnormal phenotypes. Here we discuss non-genetic causes of phenotypic heterogeneity of hepatocellular carcinoma, with an emphasis on variability of membrane protein complexes and transferred functions raising important implications for diagnosis and treatment.

## Review

### Introduction

Hepatocellular carcinoma (HCC) is the third leading cause of cancer mortality worldwide and a significant increase in the incidence of HCC through the last two decades has been observed [1]. There are two prominent features in the development of HCC. First, 90% of HCCs have chromosomal abnormalities and, second, the great majority of these tumors, regardless of aetiology, develop in cirrhotic livers, which are characterized by destruction of the hepatic lobular architecture and its replacement by nodules containing proliferative hepatocytes, in the presence of chronic inflammation and fibrosis [2].

A seminal feature of hepatocellular carcinoma is the ability to produce multiple subpopulations of cells with diverse genetic, biochemical and immunological characteristics [3,4]. How this heterogeneity emerges and how it is maintained is not clear [5,6]. Fluctuations in single cells can be masked or completely misrepresented when cell populations are analyzed. Therefore, intra-tumor heterogeneity may foster tumor evolution and adaptation and hinder personalized-medicine strategies that depend on results from imaging procedures or single tumor-biopsy samples [7,8]. Along these lines, it has become exceedingly apparent that the utility of measurements based on the analysis of bulk tumors is limited by intra-tumor genetic and epigenetic heterogeneity, as characteristics of the most abundant cell type might not necessarily predict the properties of the whole cell populations [8]. Indeed, this aspect is supported by a recent report describing the presence of distinct diagnostic signatures derived from different biopsies of the same tumor [9]. Yet, such non-uniformities often unveil molecular patterns that can represent mechanisms of tumor progression. More interestingly, variability among single cells in a population may arise from different responses to intrinsic and extrinsic perturbations coming from the abnormal microenvironment that may have pronounced influences on the epigenetic aspects of cells, translating into abnormal phenotypes [10]. Therefore, it is tantalizing to hypothesize that normalization of the tumor microenvironment corresponds to the normalization of cellular phenotypes, and destabilization of normal tissue organization can translate into an increased risk of genomic instability and phenotype heterogeneity [11,12]. The great interest concerning the tumor microenvironment is associated with the recognition that micro-environmental alterations are not just passive consequences of genetic evolution occurring in hepatocytes, but that they are active participants in tumorigenesis [13]. As many excellent reviews summarize progress in this area, we focus on the effects of micro-environmental alterations on the phenotypic heterogeneity of pre-malignant hepatocytes.

### Activated hepatic stellate cells

Pre-malignant hepatocytes live in a complex microenvironment that includes the extracellular matrix (ECM), diffusible growth factors and cytokines, and a variety of non-epithelial cell types, including endothelial cells, activated hepatic stellate cells (HSCs), and those that can respond to infection and injury, that is, lymphocytes, Kupffer cells-macrophages and mast cells [14,15].

HSCs are known as very important ECM-producing myofibroblasts dwelling in the cirrhotic liver and microenvironment of HCC. The activated HSCs infiltrate the stroma of liver tumors and localize around tumor sinusoids, fibrous septa and capsules [16,17]. Activated HSCs increase the production and secretion of ECM proteins, which include collagens, laminins, fibronectin and heparan-sulphate proteoglycans. In this way, HSCs have a major impact on the ECM content of the microenvironment and also may affect the overall tumor stromal behavior and *vice versa*[18]. Indeed, several studies demonstrated that transformed hepatocytes stimulate migration of HSCs in culture, as well as their production of ECM components, when co-cultured, or when HCC tumor-conditioned medium was used [19-21]. Hence, all support the concept that hepatocarcinoma cells recruit HSCs, which then promote tumor growth and local invasion [22]. The cancer cell-induced increase in ECM synthesis is mediated by transforming growth factor beta (TGF-β1), whereas proliferation of HSCs is promoted by platelet-derived growth factor (PDGF) [23,24]. This interaction, between HCC and HSCs, is bidirectional since HSCs, in turn, stimulate hepatocarcinoma cell proliferation and inhibit their apoptosis to increase the population of cancer cells [25]. Proliferation of hepatocytes is mediated by factors secreted by activated HSCs, such as insulin-like growth factor I (IGF-I), transforming growth factor alpha (TGF-α), hepatocyte growth factor (HGF) and other inflammatory cytokines [26,27]. Accumulating evidence indicates and points to an important and major influence of activated HSC on HCC development and progression and, hence, the therapeutic inhibition of activated HSCs should be taken into account when treating HCC [28].

### Premalignant and cancerous hepatocytes

#### Hepatocyte plasma membrane microdomains, the tetraspanin paradigm

**Figure 1:**
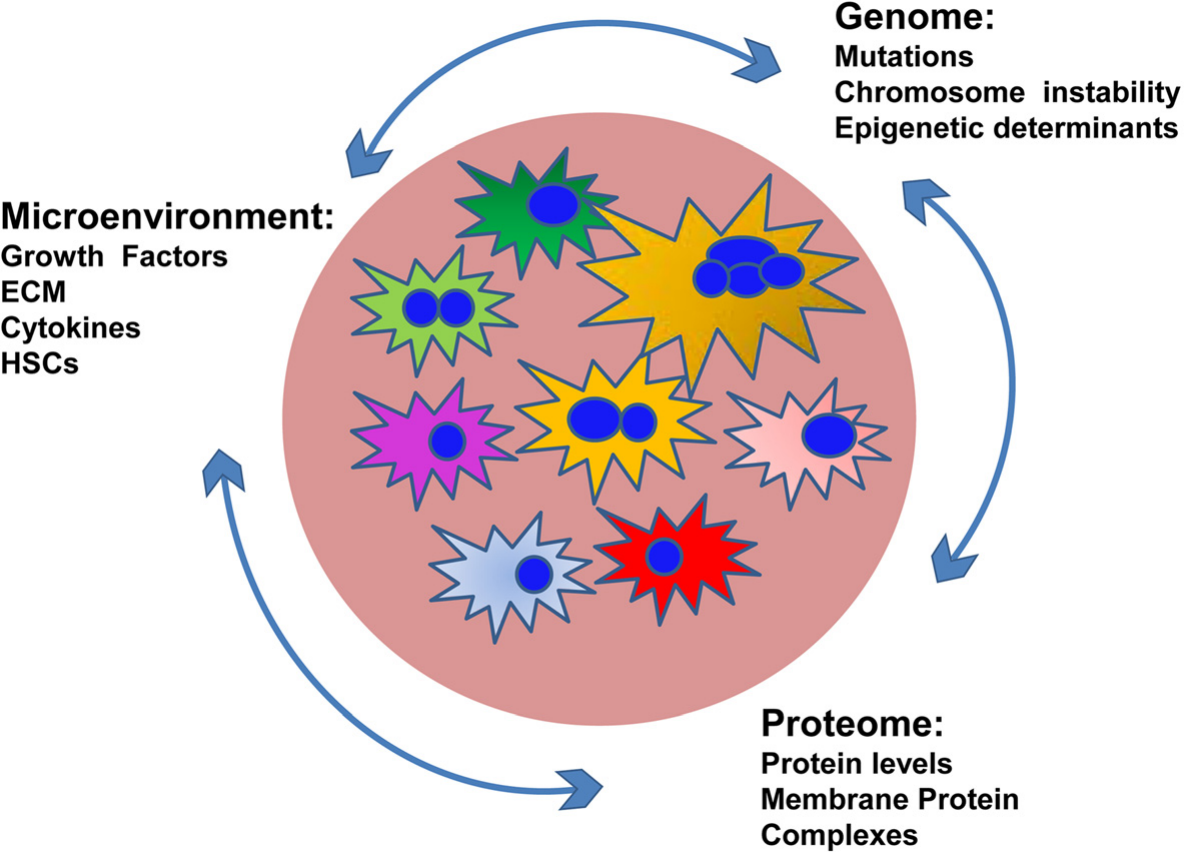
**Interaction among factors that determine phenotypic heterogeneity in HCC.** Combinations of environmental, genomic and proteomic variation can cause heterogeneity in an initially homogenous population of pre-malignant hepatocytes.

It has long been recognized that differences from one cell to the next can arise through variation in the extracellular environment or from genomic alteration. Only recently it has become clear that plasma membrane protein fluctuations can also have profound effects on phenotype. These fluctuations cause genetically identical cells to vary significantly in their responsiveness to stimuli of the fibrotic microenvironment (Figure 1).

The spatial organization of plasma membrane components in discrete microdomains is thought to be a key factor in the generation of distinct signal inputs or outputs [29]. Dynamic microdomains have important implications for understanding how signaling complexes are assembled and disassembled in response to ECM stimuli; some components of these signaling complexes might reside permanently in these microdomains, but others could have extremely transient interactions [30].

Tetraspanins are transmembrane proteins defined by small and large outer loops, short N-terminal and C-terminal tails with four transmembrane domains. They form complexes termed tetraspanin-enriched microdomains (TEMs) by interacting with other tetraspanins and with a variety of transmembrane and cytosolic proteins that are required for their function [31]. Several tetraspanin molecules have been identified and implicated in the regulation of cell proliferation, cell migration and cell fusion [32]. The most important partners are integrins, particularly α3β1, α4β1, α6β1 and α6β4, intracellular associated heterotrimeric G proteins, proteases, immunoglobulin superfamily members and cytosolic signal transduction molecules [31]. The repertoire of tetraspanins differs between cancer cell types; therefore, a complete characterization of tetraspanin-associated proteins and functions is difficult to accomplish and may not be generalized. Nevertheless, in the majority of cancer cell types, including HCC, a characteristic feature is the evident presence of integrins, signaling proteins and proteases as important components of these domains [33].

#### Tetraspanin CD81

Tetraspanin CD81 was identified originally as the target of an anti-proliferative antibody (TAPA-1) that inhibited *in vitro* cellular proliferation [34]. CD81 is involved in a broad range of cellular functions as revealed by the binding of monoclonal antibodies. The antibodies evoke their effect by mimicking a natural ligand or by altering the interactions between CD81 and its associated proteins. Although the protein is widely expressed, its levels within a single tissue vary in response to cellular activation. An important feature of tetraspanin CD81 is its ability to associate with itself forming homodimers and with various other receptors into membrane microdomains. Up-regulation of CD81 in pre-malignant hepatocytes can contribute to reorganizing the plasma membrane in domains where signaling proteins can be recruited [35]. CD81 regulation of proliferation is positively associated with activation of the extracellular signal-regulated kinase 1/2 (ERK1/2)/MAPK pathway. CD81 overexpression can activate ERK1/2 while promoting proliferation [35]. Importantly, CD81 induces reorganization of the plasma membrane amplifying the instability of pre-malignant hepatocytes and enhancing their neoplastic progression. Therefore, phenotype heterogeneity could be influenced primarily by a fluctuation of a single protein and associated factors organized in discrete plasma membrane domains. These membrane microdomains represent versatile devices for compartmentalizing different signaling functions. In the non-activated state they float freely, carrying a few passenger proteins, but, when activated, they coalesce to form larger platforms where proteins meet to transfer functions in signaling, processing and transport [36].

When the hepatocytes progress in their transformation, the tendency of CD81 expression is to be lost, as revealed by two clinical studies showing a decreased or absent CD81 expression, particularly in metastatic tissues [37,38]. HCC cells re-expressing CD81 are still capable of proliferating and producing the principal tumor when injected into the liver of nude rats; however, they contain a defective faculty to produce tumors in distant parts of the liver [39]. These findings strengthen the vision that CD81 is a facilitator of cell proliferation and in the meantime is a negative controller/regulator of movement when expressed by the cells. This is supported by the current view that cell growth and ability to metastasize are two conditions of malignancy not necessarily overlapping [40].

#### Tetraspanin CD151

The initial evidence that CD151 promotes metastasis came from a study showing that an antibody with unknown specificity inhibited metastasis formation by a human epidermoid carcinoma cell line *in vivo*. The antibody was found to recognize CD151, and inhibit cell migration without affecting adhesion or proliferation [41]. Overexpression of CD151 is seen in many tumor types. In breast, pancreatic, colorectal and non-small-cell lung cancer, high CD151 expression is associated with a poor prognosis [33].

**Figure 2:**
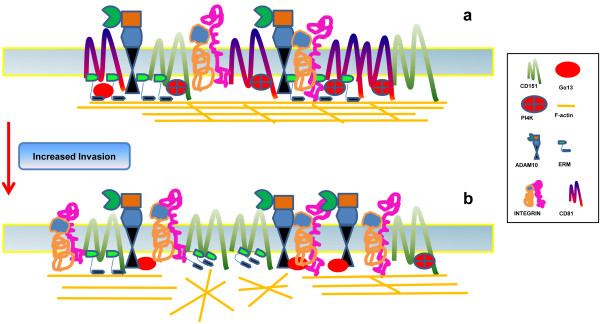
**Tetraspanin**-**enriched microdomain variation as a component of HCC progression.** The signaling pathway varies between cell types when differential TEM profiling is expressed following exposure to a cell agonist or through changes in the microenvironment. These variations in signaling can profoundly affect the tumorigenicity and metastatic properties of HCC cells. **a)** Following stimulation, CD81-associated proteins inhibit tumor cell migration, possibly through a blockade of ezrin-radixin-moesin (ERM) protein activation, inhibiting actin reorganization. **b)** Reduced expression of CD81 and up-regulation of CD151, α6β1 integrin and ADAM10 foster invasion and possibly metastases through events of actin cortex - membrane destabilization during cell motility.

Overexpression of CD151 has been associated with poor prognosis also in HCC. Some studies have indicated that CD151 overexpression promotes the metastasis/invasion of cancer cells by mediating integrin signals, while others have argued that an increased expression of CD151 contributes to activate phosphatidylinositol 3-kinase/protein kinase Akt pathway [42]. Indeed, the high expressions of CD151 and α6 integrin are major contributors to the invasion-prone phenotype of HCC. In contrast with CD81, the contribution of CD151 to HCC metastasis/invasion provides an example of the facilitator role of this tetraspanin (Figure 2). Apart from CD151, the tetraspanin TSPAN8 (previously known as CO-029, TM4SF3) has been also associated with tumor progression [43]. Overexpression of TSPAN8 is described on hepatocellular carcinomas that are poorly differentiated and prone to intrahepatic spreading [44]. Conversely, down-regulation of tetraspanin CD82/KAI1 was observed at the levels of both mRNA and protein. This was particularly pronounced in poorly differentiated HCCs. Importantly, the CD82/KAI1 level correlated inversely with intrahepatic metastases [45].

#### Differences in integrin expression and signaling within HCC

The pattern of integrins expressed by human hepatocytes is strikingly different from most other epithelial cells [46]. Normal adult hepatocytes express low levels of only three integrins: α1β1, a collagen and laminin receptor; α5β1, a fibronectin receptor; and α9β1, a tenascin receptor. In contrast, other integrin receptors, such as α2β1, α3β1, α6β1 and α6β4, are undetectable on normal hepatocytes. One of the most frequent alterations during liver carcinogenesis is the *de novo* expression of the integrin, α6β1. HCC patients characterized by multiple tumors, vascular invasion and the absence of encapsulation exhibit increased α6β1 expression [47]. In fact, the induction of α6β1 is an early event in hepatocellular carcinogenesis, and it is reasonable to consider that α6β1 contributes to hepatocarcinogenesis based on several lines of evidence [48-51]. For this reason, it is important to understand the mechanism by which the α6β1 integrin influences the function of HCC cells. One likely possibility is that α6β1-mediated activation of focal adhesion kinase (FAK) and ERK1/2 controls signaling pathways important for HCC function [52]. Both FAK and ERK1/2 are of interest because they are regulated by integrin-mediated attachment to ECM, as well as growth factor stimulation, and they control important functions of tumor cells, such as growth and migration. Another possibility suggests that overexpression of α6β1 could provide a ligand-independent growth advantage by modulating the cellular architecture or a signaling pathway required for cell growth [51].

Interestingly, the role of α3β1 integrin appears more controversial in hepatocarcinogenesis. A previous study indicated that TGF-β1 was able to induce a significant increase in the expression level of α3β1, which consecutively cooperated with TGF-β1 to induce HCC cell epithelial-mesenchymal transition (EMT) [53]. In a recent study, investigators could not confirm the previous findings when evaluating α3β1 expression in HCC tissue specimen of patients with high concentration of serum TGF-β1 levels nor could be demonstrated a significant up-regulation of α3β1 in HCC cells after 24 or 48 hours of TGF-β1 stimulation. Indeed, they find that the amplified integrin α6β1 signaling pathway is able to induce EMT of HCC cells [42].

#### ADAMs

ADAMs are multidomain proteins that contain a disintegrin and a metalloprotease domain [54]. Their metalloprotease domains can induce ectodomain shedding and cleave ECM proteins [55]. Their disintegrin and cysteine-rich domains have adhesive and fusion activities. Hence, ADAMs are poised to modulate a variety of cell-cell and cell-ECM interactions. ADAM10, a member of the ADAM family, was detected in all human HCC tissues tested by immunohistochemistry but not in normal liver tissues [56]. Moreover, CD44, a typical substrate of ADAM10 protease, was also expressed in all human HCC tissues but not in normal liver tissues. These data suggest that overexpression of ADAM10 and CD44 is a characteristic of human HCC. Specifically, ADAM10 is involved in the intramembrane proteolysis process, whereby it mediates ectodomain shedding of various membrane-bound receptors, adhesion molecules, growth factors and cytokines [57].

## Conclusions

HCC cell phenotypes are the result of the integration of inputs from genotype and environmental stimuli. Epigenetic changes that arise during tumor progression alter and diversify cellular phenotypes, posing a major obstacle to the understanding and clinical management of HCC. We suggest that the phenomenon of intra-tumor phenotypic heterogeneity, especially aspects that are related to clonal diversity, deserve to be recognized and accounted for during the analysis of HCC tumor, building of experimental models and design of therapeutic approaches.

The dominance of gene-centric views has been challenged with the rapid development of research establishing that because tumors contain phenotypically distinct populations of both tumor and stromal cells that interact in a dynamic and reciprocal manner, these interactions are likely to result in the emergence of different proteome profiling. This aspect creates significant problems in employing therapeutic procedures in which micro-environmental changes make a procedure inefficient and in some regions of the HCC a therapeutic result may not be achieved. This inequality of therapy gives HCC cells time to develop resistant phenotypes. In addition, components of the microenvironment can actively protect tumor cells from treatment through secreted factors and cell contact-mediated pro-survival stimuli. Heterogeneity in the tumor microenvironment translates into heterogeneity of tumor cell phenotypes, and so some tumor cells might be intrinsically less sensitive to the therapy. Intra-tumor heterogeneity, associated with heterogeneous protein function, may promote HCC progression through Darwinian selection.

## Abbreviations

ADAM: A-disintegrin and metalloprotease; Akt: Protein kinase B; ECM: Extracellular matrix; EMT: Epithelial-mesenchymal transition; ERK: Extracellular regulated protein kinase; ERM: ezrin-radixin-moesin; FAK: Focal adhesion kinase; HCC: Hepatocellular carcinoma; HGF: Hepatocyte growth factor; HSCs: Hepatic stellate cells; IGF-I: Insulin-like growth factor I; MAPK: Mitogen-activated protein kinase; PDGF: Platelet-derived growth factor; TEMs: Tetraspanin-enriched microdomains; TGF-α: Transforming growth factor-alpha; TGF-β1: Transforming growth factor-beta.

## Competing interests

The authors declare that they have no competing interests.

## Author’s contributions

KR and VC wrote the manuscript. VC finalized the manuscript and organized the figures. Both authors read and approved the final manuscript.
